# Biosynthesis of Phytocannabinoids and Structural Insights: A Review

**DOI:** 10.3390/metabo13030442

**Published:** 2023-03-17

**Authors:** Rasiravathanahalli Kaveriyappan Govindarajan, Awdhesh Kumar Mishra, Kiu-Hyung Cho, Ki-Hyun Kim, Kyoung Mi Yoon, Kwang-Hyun Baek

**Affiliations:** 1 Department of Biotechnology, Yeungnam University, Gyeongsan 38541, Gyeongbuk, Republic of Korea; 2 Gyeongbuk Institute for Bioindustry, Andong 36618, Gyeongbuk, Republic of Korea

**Keywords:** *Cannabis sativa* L., phytocannabinoids, biosynthesis pathways, structural activity, industrial application

## Abstract

Cannabis belongs to the family Cannabaceae, and phytocannabinoids are produced by the *Cannabis sativa* L. plant. A long-standing debate regarding the plant is whether it contains one or more species. Phytocannabinoids are bioactive natural products found in flowers, seeds, and fruits. They can be beneficial for treating human diseases (such as multiple sclerosis, neurodegenerative diseases, epilepsy, and pain), the cellular metabolic process, and regulating biological function systems. In addition, several phytocannabinoids are used in various therapeutic and pharmaceutical applications. This study provides an overview of the different sources of phytocannabinoids; further, the biosynthesis of bioactive compounds involving various pathways is elucidated. The structural classification of phytocannabinoids is based on their decorated resorcinol core and the bioactivities of naturally occurring cannabinoids. Furthermore, phytocannabinoids have been studied in terms of their role in animal models and antimicrobial activity against bacteria and fungi; further, they show potential for therapeutic applications and are used in treating various human diseases. Overall, this review can help deepen the current understanding of the role of biotechnological approaches and the importance of phytocannabinoids in different industrial applications.

## 1. Introduction

Cannabis, scientifically known as *Cannabis sativa* L., is a genus within the family Cannabaceae; it is a wind-pollinated, dioecious herb (i.e., the male and female reproductive structures are on separate plants), although monoecious plants can occur in some populations [1]. Additionally, the species *C. sativa* L. is a potential source of fiber, food, oil, and protein [2]. The discovery of the curative virtues of plants is attributed to Chinese research dating back to 10,000 BC [3], and the first phytocannabinoids were isolated from *C. sativa* L. [4]. Phytocannabinoids are structurally diverse, naturally obtained compounds produced in several secondary metabolite plants, and they have a long, controversial history of use and abuse [4,5]. Cannabis has several varieties suitable for various purposes; hence, it has been widely used in industrial, ornamental, nutritional, recreational, and pharmaceutical applications and herbal medicine [6]. Cannabinoids are predominantly insoluble in water but soluble in alcohol and other nonpolar solvents [7]. According to the recent literature, over 200 phytocannabinoids have been identified in the cannabis plant [8].

*Cannabis sativa* L. subspecies are plants that contain a large variety of secondary metabolites, including phytocannabinoids, terpenoids, and flavonoids, which have profound anti-microbial activities, in addition to possessing anti-inflammatory, anti-oxidative, and neuromodulatory properties [8]. They are classified into different subclasses according to their chemical structure [9]. Cannabidiol (CBD), ∆^9^-tetrahydrocannabinol (∆^9^-THC), cannabigerol (CBG), and cannabinol (CBN) are the most studied [9]. During this research, numerous countries stopped growing plants [10]. According to the World Health Organization (WHO), an estimated 80% of people in developed countries rely on herbal medicine to meet their basic health needs and treat various diseases [11].

Nonetheless, these plants are perceived as criminal and unacceptable to communities, as most consumers cannot differentiate between psychoactive and non-psychoactive cannabis plants. By contrast, the use of cannabis plants with low levels of THC in medicine and foods is based on various potentially beneficial cannabinoid compounds [11], including CBD [12]. This is synthesized by postsynaptic neurons and acts as a retrograde messenger molecule for neurotransmitter release from CB_1_ expression, which is found in areas of the human brain with high densities of cannabinoid receptors (CBrs) [13]. The endocannabinoid system also plays a key role in neural and molecular control mechanisms. In fact, the endocannabinoid system plays a key role in the normal physiological functions of the gastrointestinal tract, including motility, gut–brain-mediated fat intake, hunger signaling, inflammation, and intestinal permeability [14,15]. Furthermore, cannabis fiber has been used in harmless, inexpensive, eco-friendly, biodegradable raw materials (all derived from plant-based products) to produce cellulose, biofuels, and bioethanol, such as paper towels, paper plates, ready-made clothes, and even natural fiber shoes [16]. It demonstrates its capability for sustainable agricultural production, aiming to protect the soil and environment through lack of water consumption in the production of hemp fibers, and the ability to grow without the need for fertilizer and pesticides [16]. Despite the ornamental feasibility of cannabis, its suitability for the ornamental industry remains largely unrecognized, and studies tend to focus primarily on botany, cultivation, propagation, and other agricultural aspects of cannabis [14]. Numerous developed countries, such as the UK, USA, Canada, and the Netherlands, have supported the production of cannabis [17]. Edible usage is classified into medicinal and recreational purposes [18]. However, the permitted amount of CBD per serving should not exceed 20 mg; and each package sold should not exceed 600 mg of CBD [15] and should contain less than 0.001% THC [18]. Whereas, in the USA, edibles should contain less than 0.3% THC and have CBD at levels of cannabis edibles for medicinal purposes [17]. Based on 2017 research, the total hemp wholesale market was valued at more than 700 million dollars in the USA. Despite the decline in the agriculture of cannabis owing to existing legal restrictions, standard procedures have been established to encourage the natural production of fiber from these plant species [18].

Moreover, these low levels of psychoactive components are what make pharmaceutical industries bet largely on hemp to obtain the non-psychoactive cannabinoid cannabidiol (CBD), which has shown a high therapeutic value in numerous diseases. Therefore, among cannabis products for skin care, CBD oil with high therapeutic potential and without undesirable psychotropic effects has been extracted from leaves [18]. Numerous chemicals are produced in hemp through secondary metabolism. They include cannabinoids, terpenes, and phenolic compounds [19]; these will be further described in the next sections. Although the pharmacological properties of cannabinoids have been extensively studied and are the most recognized hemp bioactives, other components have also been associated with potent health-promoting properties [19]. At the same time, these products are free of chemicals and toxins that are used in most synthetic cosmetics. CBD has a wide spectrum of biological activity, such as antioxidant and anti-inflammatory activity, and therefore can be used for the treatment of diseases associated with redox imbalance and inflammation. CBD can be used for the treatment of diabetes-related cardiomyopathy, (including stroke, arrhythmia, and hypertension), cancer, anxiety, psychosis, epilepsy, neurodegenerative disease, musculoskeletal pain, and skin disease [20]. A mixture of cannabinoids may produce tattoo ink with a low risk of infection and inflammation [21]. Certain natural plants possess at least some beneficial properties in mitigating biotic and abiotic stress [22]. However, the genetic mechanism of trichome development and, subsequently, the cannabinoid content have not been updated, and this remains a significant factor in cannabinoid production [23,24]. In the past several decades, a lack of knowledge of hemp and marijuana has reduced the growth of plants. Advanced research has focused primarily on the benefits of plants and other phytocannabinoids with weak or no psychoactivity with promise as therapeutic agents in human health. Thus, the terminology and important tools must be clarified, and clear descriptions must be elucidated for differentiating between therapeutic and other supplements derived from cannabis as technology advances [25]. This review presents the various sources of cannabis, the biosynthesis of phytocannabinoid pathways, the structures of the enzymes in the pathways, and the properties of phytocannabinoids that are important to animals and humans. Phytocannabinoids exhibit antimicrobial activities against bacteria and fungi [26]. Furthermore, the uses of cannabis are economically significant, owing to the various food industry applications and the development of therapeutic and pharmacological applications of phytocannabinoids. Therefore, the challenges associated with phytocannabinoids in advanced biotechnology and their most significant commercial interest is elucidated in this review.

## 2. Sources of Phytocannabinoids in *C. sativa* L.

Cannabis has many trichomes, which are collected from the protuberances, close to the plant’s leaves, flowers, seeds, and other important sections. These trichomes are found in *C. sativa* L., and they are divided into various types of glandular and non-glandular structures [27]. The secretory trichome’s numerous biologically active compounds are synthesized, which take the form of protuberances and cover the plant’s leaves and stems. Over 200 compounds with diverse biological activities, including flavonoids, terpenoids, and cannabinoids, have been confirmed in hemp. Many of the current common hemp varieties are individually similar and closely related [28]. The phytocannabinoid compound ∆^9^-THC is found in farming, some construction biomaterials, and certain textile industries and includes a negligible amount of the psychoactive cannabinoid compound [29]. Its secondary metabolites include phytocannabinoids, flavonoids, terpenoids, lignans, and alkaloids [29]. Phytocannabinoids are bioactive terpenoids that are thought to be exclusive to *C. sativa* L. [30]. They play a role in a variety of physiological and pathophysiological processes. Many of these effects are mediated by two G-protein-coupled receptors (GPCRs) in the CNS and are found in particularly high levels in the neocortex, basal ganglia, cerebellum, and brainstem [31]. Interestingly, CB_1_ receptors are highly enriched at presynaptic and axonal compartments and restrict their function to sites of synaptic activity [32]. The CB_2_ receptors exhibit a more defined pattern of expression in the brain than CB_1_ receptors and are found predominantly in cells and tissue of the immune system [33].

## 3. Structures of Phytocannabinoids

**Figure 1:**
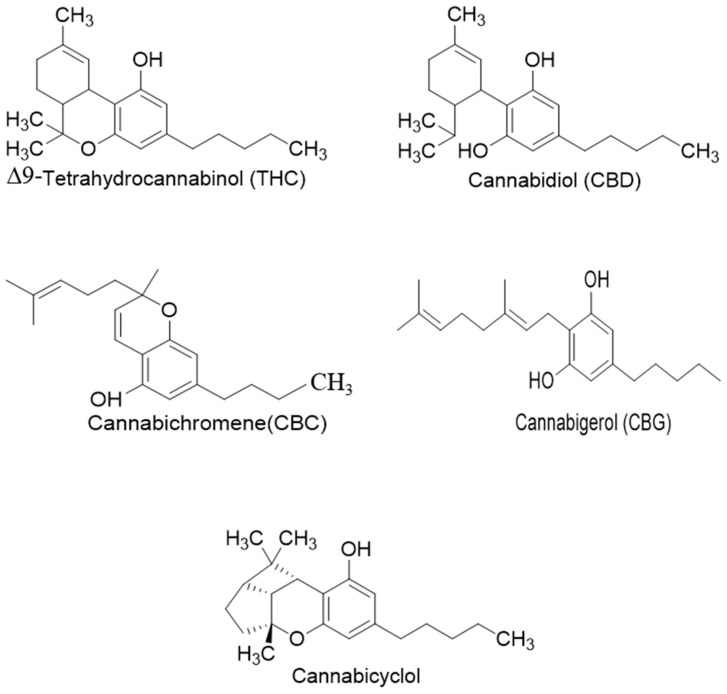
Structure of phytocannabinoids.

Phytocannabinoids have been found in different plant species of cannabis, including *Echinacea purpurea*, *E. angustifolia*, *E. pallida*, Acmella oleracea, Helichrysum umbraculigerum, and *Radula marginata* [12,33]. A group of C_21_ and C_22_ carboxylated forms of terpenophenolic compounds exhibit binding affinity at cannabinoid receptors. However, phytocannabinoid synthesis involves several structures of chemical compounds, such as THC, CBD, CBG, CBC, cannabicyclol (CBL), and cannabidiol type (CBND). Phytocannabinoids are represented by several motifs composed of various moieties: the isoprenyl residue, resorcinol core, and alkyl group [34] (Figure 1). Δ^9^-tetrahydrocannabinol (Δ^9^-THC) and cannabidiol (CBD) are the most abundant compounds in cannabis plants and are central to their therapeutic application [35,36]. There is limited access to low-THC/high-CBD products (i.e., products with low levels of THC and high levels of CBD) [36]. Of the conditions that allow for some access to cannabis compounds, cancer, HIV, multiple sclerosis, glaucoma, seizures/epilepsy, and pain are among the most recognized qualifying ailments [37]. Medical cannabis is also used for the treatment of any illness for which the drug provides relief for the individual. In the last few years, numerous studies have investigated natural and synthetic cannabinoids for targeting and killing tumors, to the point of collecting overwhelming evidence to suggest that cannabinoids can be used as adjuvant agents for the treatment of cancer [12].

## 4. Biosynthesis and Pathway of Phytocannabinoids 

Phytocannabinoids are stored in glandular trichomes, located all over the aerial part of the plant; the root surface and root tissue do not produce phytocannabinoids [34]. Moreover, plants grown outdoors are exposed to a fair amount of ultraviolet UV-A and UV-B, and exposure to UV rays before harvest causes the plant to produce more THC compounds [35]. Adaptive responses to changing UV-B conditions also play an important role in plant–herbivore interactions. UVB-mediated changes in plant architecture, physiology, and/or chemistry can alter herbivorous arthropods’ performance and preference. In most cases, these UV-B-mediated induced physiological changes lead to the reinforcement of plant defenses. For example, increased production of UV-B-protective secondary metabolites and/or the reinforcement of plant cell walls induced by UV-B was proposed to affect plant colonization by herbivorous arthropods [35]. The pathways are split between different cell types and organelles, the cytoplasm of the gland cells, plastids, and storage of the extracellular cavity [12]. Many cannabinoids are formed in correlation with increased moisture, heat, temperature, and abiotic stress conditions, as well as low soil moisture content. The study demonstrated the potential of mineral nutrition to regulate cannabinoid metabolism and optimize the pharmacological quality of potassium, an essential plant nutrient that is required by plants in relatively high amounts and takes part in many key physiological processes [30]. Processes affected by K supply include stomatal regulation, protein synthesis, photosynthesis, enzyme activation, osmoregulation, and the uptake and accumulation of other essential cations such as Ca and Mg [36]. In addition to its involvement in the regulation of plant primary metabolism, K is known to have a considerable impact on the secondary metabolism of plants and is therefore considered a quality element [35]. As K is known to affect a wide range of secondary metabolites in plants, including phenolic compounds, flavonoids, carotenoids, and organic acids, we hypothesized that it has the potential to regulate the production of medical compounds in *C. sativa* [36]. UV rays at 320 nm provide an evolutionary benefit by acting as sunlight to flowering plants, thus resulting in the production of a greater number of phytocannabinoids conveying different biological benefits and activities; this represents a crucial light signal to which plants can respond and develop specific photomorphogenic responses [37,38]. Among these responses, changes in the morphology, physiology, and production of secondary metabolites are commonly described. The main psychoactive constituent in cannabis is THC, and the main non-psychoactive compound is CBD, which accumulates in glandular trichomes and is obtained from plant tissue, flowers, and a high density of plant constituents [38]. The cannabinoid pathways involve the hexanoyl-CoA produced by an acyl-activating enzyme (*Cs*AAE1) derived from hexanoic acid through the fatty acid biosynthesis pathway [39]. Hereafter, type III polyketide synthase olivetol synthase elongates toward hexanoyl-CoA using three units of malonyl-CoA and has characteristics of another type III polyketide synthase (PKSs) in flowering plants; this method of production requires significant resources and is an unsustainable avenue for harvesting cannabinoids, given its recent rapid expansion in demand worldwide [40]. Instead, another enzyme, olivetolic acid cyclase (OAC), catalyzes the necessary C_2_ to C_7_ aldol condensation reaction to produce olivetolic acid (OLA) (Table 1).

**Figure 2:**
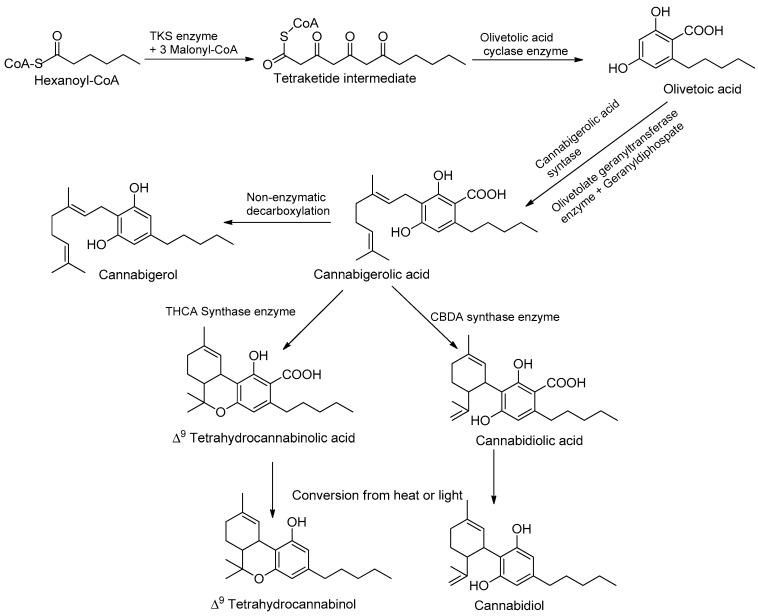
Cannabinoid biosynthetic pathway, leading to the two major phytocannabinoids, THC and CBD.

A native prenyltransferase from *C. sativa* L. that completes the step was identified as CsPT1 (or CsPT4), or geranylpyrophosphate: olivetolate geranyltransferase (GOT), in 1998. Geranylpyrophosphate (GPP) is assumed to be supplied through the 2-C-methyl-D-erythritol-4-phosphate (MEPs) pathways owing to cannabinoid synthases [47]. The pathway can be separated into three distinct functional activities, and the polyketide pathways can produce OLAs and isoprenoids and can produce cannabigerolic acid (CBGAs), as the first cannabinoids in collaboration with the prenyltransferase [48] (Figure 2). Recent work has focused on capitate stalked glandular (CSG) trichomes, which consist of two parts—a nearly spherical resin head (gland head) atop a multicellular stalk. The resin head incorporates a rosette of secretory disk cells at its base, covered by a thin, distensible sheath or cuticle. Cannabinoids and terpenoids accumulate in a secretory cavity between the disk cells and the cuticle [49]. Disk cells also secrete biosynthetic enzymes, such as THCA synthase, into the secretory cavity [46]. The pathway, with key chemical structures, is illustrated in Figure 2. See the chapter by Supaart Sirikantaramas et al. [49] for an elaboration. Cannabinoid content differs in terms of quantity and quality. Quantity and quality have different modes of inheritance [15]. Cannabinoid quantity (dry weight percentage) is polygenic and influenced by environmental factors. This is achieved through differential cyclization of the C10 moiety from GPP via the actions of independent synthases [49]. The discussion on synthetic biology approaches will be organized according to these individual enzymatic steps in the pathways, along with structural and mechanistic insights, to facilitate the subsequent framing of strategies for producing novel cannabinoids [50].

## 5. Structural Analysis of the Enzymes THCA and CBDA Synthase

**Figure 3:**
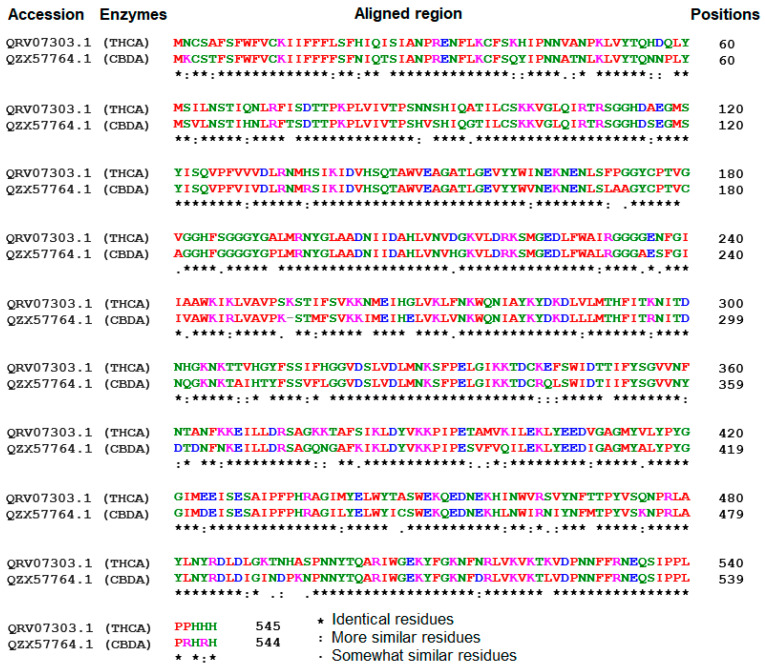
Sequence alignment among tetrahydrocannabinolic acid (THCA) and Cannabidiolic acid (CBDA) synthases using the Clustal Omega alignment server.

**Figure 4:**
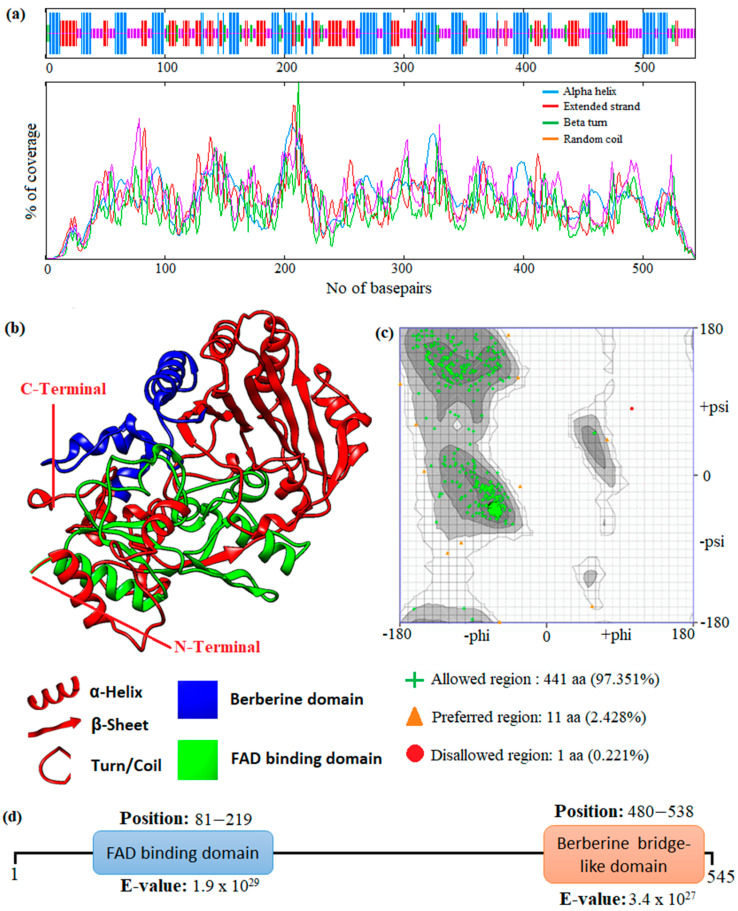
Prediction of the 1D, 2D, and 3D structural profile of Tetrahydrocannabinolic acid synthase. (**a**) Secondary structural elements, (**b**) Predicted 3D model, (**c**) Validated model, and (**d**) Predicted motif and domain regions.

**Figure 5:**
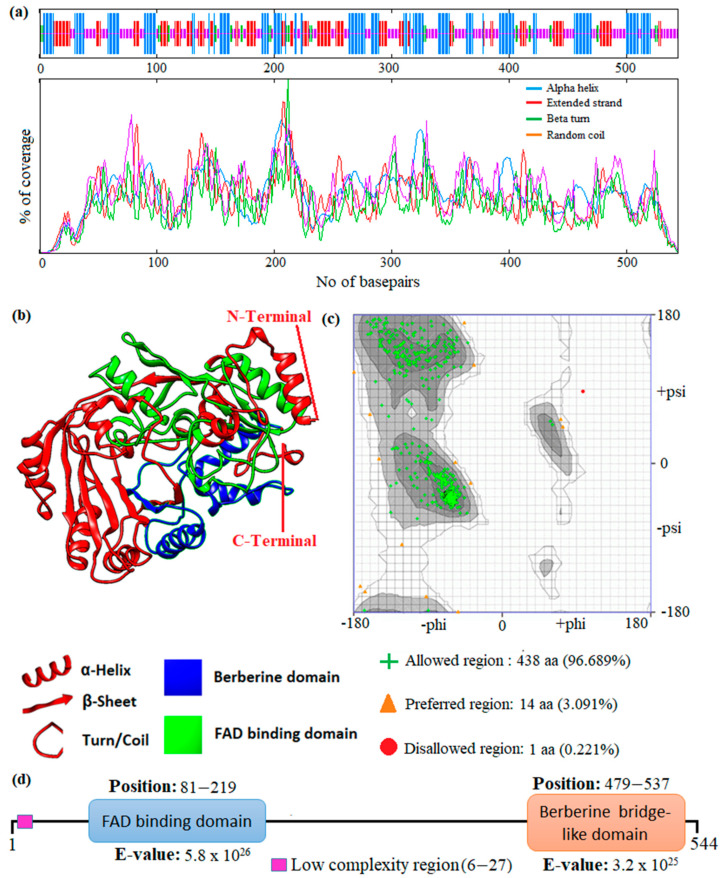
Prediction of the 1D, 2D, and 3D structural profile of Cannabidiolic acid synthase. (**a**) Secondary structural elements, (**b**) Predicted 3D model, (**c**) Validated model, and (**d**) Predicted motif and domain regions.

In this study, an in silico approach was used to perform structural analysis (1D, 2D, and 3D) of enzymes such as THC acid and CBD acid synthase. The primary compositions of the nucleotide and protein sequences were predicted using BioEdit V 7.2.6.1 [51] software, as represented in Supplementary Table S1. The pairwise sequence alignment of THC acid and CBD acid synthase was performed using the Clustal Omega server (https://www.ebi.ac.uk/Tools/msa/clustalo/ accessed on 15 February 2023). The alignment shows 90.6% of sequences are similar to one another, as depicted in Figure 3. Whereas the secondary structural elements of target enzymes were predicted using the (SOPMA) server (https://npsa-prabi.ibcp.fr/NPSA/npsa_sopma.html/accessed on 15 February 2023). The results revealed that a maximum number of residues formed a random coil, which determines the stability of enzymes. Further, 170 amino acid residues were found to be involved in alpha-helix and 132 for extended strand formation, as shown in Figure 4a and Figure 5a.

Protein 3D structures were modeled with the Swiss Model [52], and the predicted structure was confirmed with the Ramachandran Plot server (https://zlab.umassmed.edu/bu/rama/accessed on 15 February 2023). The predicted model exhibited 100% similarity with the THCA enzymes and 83.72% for CBDAs, using the protein data bank (PDB) template 3VTE, as depicted in Figure 4b and Figure 5b, respectively. According to the structural validation results (Ramachandran plot), 441 and 438 amino acid residues were found in the allowed region, which occupies 97.351% and 96.689% for THCA and CBDA synthase, as represented in Figure 4c and Figure 5c, respectively. Our target enzyme’s motif and domain region were predicted using the smart server and Conserved Domain Database NCBI-CDD [53] search. The prediction revealed that two major domains, the FAD binding domain at positions 81–219 and Berberine bridge-like domain at positions 480–538, were found in both THCA and CBDA enzymes (Figure 4d and Figure 5d, respectively). Interestingly, a low-complexity region (repeats of single amino acids) was found in position 6–27 bases only in CBD [54]. In the 1960s and 1970s, numerous plausible hypotheses were advanced regarding the biosynthesis of THCA; however, they all were lacking experimental support. THCA was thought to arise from CBDA through cyclization. However, the reaction conditions of the transformation do differ from those present during natural biosynthesis in the plants; moreover, isomerase activity, which would be necessarily responsible for the conversion of CBDA into THCA, has never been detected in any enzyme assays using crude *C. sativa* enzyme extracts. Current thinking suggests it comes from CBGA instead, by either tetrahydrocannabinolic acid synthase (THCAs) or cannabidiolic acid synthase (CBDAs), both members of the p-cresol methyl-hydroxylase super family.

## 6. Bioactivities of Phytocannabinoids in Animals and Humans

Phytocannabinoids convey various biologically beneficial effects and properties; thus, they have been used as therapeutics since antiquity [54]. Further, they are linked to the modulation of the endocannabinoid mechanism and the class of receptors CB_1_ and CB_2_ distributed in various cells [55]. Additionally, some studies have reported that several phytocannabinoids exhibit potent antimicrobial activity against bacterial and fungal systems, and thus they can be used as antibiotics [55]. Moreover, phytocannabinoids might have therapeutic potential have antifungal properties, such as *Phompsis ganjae* [4], *Stachybotrys bisbyi*, *Fusarium oxysporum* [36], and *Saccharomyces cerevisiae* [37]. Their use in various industries is increasing worldwide and will likely increase in the next 10 years [56,57]. The endocannabinoid system in animals exposed to these contributes to the regulation of functions (such as learning and memory, emotional processing, sleep, temperature, diverse brain functions, and addiction) and cellular metabolic processes (such as glycolysis, lipolysis, and energy balance (Table 2). 

Plant-based products found in various natural agro-industrial processes demonstrate their potential in enhancing animal and meat production and extension. This plant industry can produce seeds, leaves, seed oil, and cakes, and it is a valuable source of animal feed that is rich in protein for ruminant diets [66]. However, research on the bioavailability and leading bioactive compounds of cannabis species in ruminants, as well as the psychoactive effects of THC in humans, is limited [67]. The biotechnological use of phytocannabinoids can be enhanced through agro-industrial approaches to understand their role in medication. Moreover, apart from *C. sativa*, *Nicotiana benthamiana* also emerged as the favorable heterologous host to produce phytocannabinoids; in recent research, it was found to be a major biotechnological tool for phytocannabinoid production and for inducing genetic modification [68]. To achieve this, a biotechnological approach should be utilized to gain more knowledge on the pathways involved in phytocannabinoid production [69].

However, there is a lack of information on the bio-efficacy and bioactivity of cannabidiol in the meat milieu [70]; pilot clinical studies on animal models support the application of cannabidiol in inflammatory-based skin diseases. Additionally, non-psychotropic phytocannabinoids are used in the treatment of seborrheic dermatitis, and a recent national survey showed that among current adult users, 10.5 to 46.6% reported using cannabis solely for medical uses [71]. Of the states that allow for some access to cannabis compounds, cancer, HIV/AIDS, multiple sclerosis, glaucoma, seizures/epilepsy, and pain are among the most recognized qualifying ailments. CB_1_ (cannabinoid receptor type 1, first cloned in 1990) mRNA is highly expressed in the human brain [70]. It is also found in the heart, lungs, ovary, adrenal gland, thymus, bone marrow, and uterus. CB_1_ is also widely distributed inside non-neuronal tissues and inside various cells and tissues and co-exists with other CBRs [72]. CB_1_ is found inside the brain and displays high protein density and expression inside brain parts such as the cerebellar molecular layer, substantia nigra pars reticulate, globus pallidus external and internal, olfactory bulb, olfactory nucleus (anterior), hippocampus, and layers II–III, Va and VI of the cerebral cortexes [73]. Additionally, in humans, the highest CB_1_ level is found inside the cingulated gyrus, motor cortices, and frontal secondary somatosensory. CB_1_ is found in moderate levels inside the hypothalamus and ventral striatum and low levels in the brainstem; other respiratory control centers lack CB1. Moreover, this is the main reason phytocannabinoids have a low effect on respiratory and cardiovascular activities [74]. G-protein-coupled receptors (GPR) such as GPR18 and GPR55 serve as potent phytocannabinoid receptors. GPR18 is a “deorphanized” receptor present in cells and tissues of the thymus, spleen, small intestine, lymph nodes, leukocytes, and gametes [73]. Phytocannabinoids exerts strong therapeutic potential in humans assessed by the meta-analysis of several clinical trials; they possess a strong effect on health conditions such as nausea, vomiting, insomnia (sleeping disorder), depression, anxiety, paraplegia (paralyzes in a lower limb due to spinal cord injury), and psychosis (emotional metal disorder) and provide appetite stimulation in response to several syndromes [70]. Hemp predominantly produces bioactive compounds; it has resin glands with low THC and CBD activity. The antibacterial activity of these byproducts can be attributed to the terpenes and polyphenol compounds; phytocannabinoids are the main active compounds responsible for the antibacterial biocidal activity [72]. Another bibenzyl cis-THC, (−)-*cis*-perrottetinene (*cis*-PET), was isolated from the liverwort *Radula perrottetii* [72,73]. The cis configuration in the cyclohexene ring in cis-PET is comparable with ∆^9^-trans-THC. PET resembles ∆^9^-THC in its 3D shape and can bind to many of the same cannabinoid receptors (CBRs) as ∆^9^-THC. Moreover, research data on additives and the synergic and antagonistic activities of bioactive compounds are important to promote their use in feed as well as their industrial importance [73]. In addition, the cannabis industry is expected to grow much faster than expected, reaching USD 57 billion by 2027, with North America having the largest group of cannabis buyers; the global market is expected to cover at least 67% of the expenditure, and medicinal marijuana the remaining 33%. Other developed countries are expected to contribute to the medicinal cannabis market, thus making it the largest in the world. Several common diseases in several mammal species can be treated with phytocannabinoids, which can represent a translational improvement in bioactive phytocannabinoids; however, legislation must be passed to enable this [74,75].

## 7. Antimicrobial Activity of Phytocannabinoids

Antimicrobial resistance threatens the viability of modern medicine, which is largely dependent on the successful prevention and treatment of bacterial infections. Unfortunately, there are few new therapeutics [74]. A total of 150 phytocannabinoid compounds have been identified [75]. In addition, phytocannabinoids synthesized from cannabis and various *Rhododendron* sp. produce cannabinoids of the CBC type, whereas flowering plants, including *Helichrysum umbraculigerum* Less, *Glycyrrhiza foetida*, and *Amorpha fruticosa*, contain highly bioactive compounds with a cannabinoid backbone, carry an aralkyl side chain, and contain a resorcinol core [15]. Other cannabinoids, including bibenzyl analogs of ∆^9^-THC, have been isolated from liverworts, including *Radula perrottetii* [76]. Cannabinoids have been found in fungal species, including cannabiorchromenic acid from *Cylindrocarpon oidium* [77], which produces a considerable amount of biomass in a short duration. Compounds and hemp fibers have high amounts of lignin, cellulose, and other metabolites, which makes them suitable for animal feed [78]. Cannabidiol (CBD) is the major non-psychoactive component isolated from *C. sativa* and has been associated with multiple and potential biological activities, especially anxiolytic, antipsychotic, anti-inflammatory, analgesic, antioxidant, and neuroprotective properties [79]. The potential uses of cannabidiol in antibacterial therapies has recently emerged [80]. Since the 1950s, *C. sativa*-based preparations have also been investigated for their antibacterial activity [80]. Nonetheless, few studies have described the antibacterial activity of ultrapure CBD; CBD has a bacteriostatic as well as bactericidal effect against various Gram-positive bacterial species, such as *Staphylococcus aeruginosa*, *Methicillin-resistant S. aureus* (MRSA), and *Streptococcus faecalis*, and several fungal species, including *Aspergillus niger* and *Candida albicans* [81]. Recent research in the dental discipline suggests that CBD has a pronounced time-dependent inhibitory effect on biofilm formation as well as disrupts the mature biofilm of *Candida albicans* [82]. Antibacterial activity was tolerated owing to the nature of the prenyl moiety and its relative position compared with the pently moiety and carboxyl group [83]. Studies have reported that CBCA might exert antibacterial effects degrading the bacterial lipid membrane and altering the bacterial nucleoid [82]. Jin and Lee [81] investigated the antimicrobial and anti-lipogenic effects of hemp seed extract in HaCaT keratinocytes, as well as anti-inflammatory effects in stimulated HaCaT cells by reducing the expression of genes encoding inflammatory effects [84]. However, any potential relationship between cannabis use and microbial-induced diseases must be investigated and understood in detail [85]. Phytocannabinoids have a diverse roles in humans and exhibit antimicrobial and antibiotic activity. Apart from this, they also have some other biologically beneficial properties in mitigating biotic and abiotic stress in plants [12]. Cannabis trichomes possess phytocannabinoid in large quantities. High temperatures or herbivory cause trichome rupture and the release of the phytocannabinoid content, which protects the plant from desiccation and high-temperature stress [67]. It was also reported that phytocannabinoid production was enhanced in cannabis flowers after UV-B-induced stress. Significantly, phytocannabinoids inside oil bodies provided tolerance against several abiotic stresses, such as cold temperature, heat, excessive light, and UV radiation [71]. There is a lack of knowledge about the role of phytocannabinoids in biotic and abiotic stress mitigation. Thus, more in-depth studies are still required to understand the mechanism involved in stress tolerance via phytocannabinoids [85].

## 8. Phytocannabinoid Applications in Medicinal Therapies

The phytocannabinoids approved by the pharmaceutical industry and cannabinoids have been valuable starting compounds for the development of drugs, including Nabiximols (marketed as Sativex oral spray (2.7 mg/mL THC and 2.5 mg/mL CBD) by GW Pharmaceutical, Cambridge, UK). This drug has been approved in Canada, the USA, Europe, and several developing countries for treating muscle and neurological disorders, multiple sclerosis, and cancer [84]. Several synthetic drugs, such as cannabinoid-based drugs, have been approved for the relief of vomiting associated with cancer chemotherapy (Marinol, (Dronabinol), Solvay Pharmaceutical, and Cesamet) [85]. The numerous and versatile effects of cannabis contribute to the cannabinoid system (CBs) in different processes and help regulate several physiological and health diseases [86]. This plant treatment is a particularly valuable treatment for targeting the endocannabinoid system (eCBS); modulation by phytocannabinoids is currently being adopted to limit the biotherapeutic bioavailability of cannabis metabolites and treat a multitude of human diseases [87]. Phytocannabinoids have valuable efficacy in reducing the indicators of seizure, and several hypotheses dictate that endocannabinoid system (ECS) modulators affect neurogenesis. Where symptomatic benefit is observed, it is more commonly reflected in decreased agitation and aggression, increased appetite, sleep quality, objective mood, and pain control. Phytocannabinoids are promising candidates for treating symptoms of neurodegenerative and other diseases [88]. For example, administering CBD (30 mg/kg) intraperitoneally over 2 weeks increased hippocampal neurogenesis in rodents [89]. The effects of CBD were mediated by CB1 receptors, and with an increased level of the ECB ligand in the hippocampus with phytocannabinoid compounds, several effects on neurological factors were enhanced [90,91]. Owing to the evidence that cannabinoids are more effective at reducing nausea, in combination with the U.S. Food and Drug Administration (FDA)’s approval of dronabinol, the latter is now used therapeutically in the USA and other developed countries as a vomiting and nausea-related cancer treatment drug [92]. Additionally, phytocannabinoids have the ability to counteract anorexia, cachexia, and weight loss and act as an appetite stimulant in patients under chemotherapy [93]. Adverse effects are similar across diverse peoples, and it may be considered to mitigate these individual effects. The endocannabinoid system involved in the prominent subtype of the central nervous system (CNS) has garnered considerable attention as a potential therapeutic avenue for several pathological and neuropsychological disorders as well as neurodegenerative diseases. The possibility of patenting hemp cultivars with high yields in the pharmaceutical industry has led to the cultivation of hemp in aerobic environments, exclusively for the pharmaceutical industry.

## 9. Phytocannabinoids in Industrial Applications

Phytocannabinoids are naturally occurring cannabinoids found in the cannabis plant. Recently, numerous countries have increased their interest in the production and recreational use of marijuana as well as its tremendous medical and industrial applications. More than 100 naturally occurring compounds are produced by cannabinoids, the most abundant of which are Δ^9^-THC, CBD, terpenes, and flavonoids, which are used to treat various chronic diseases [83]. Cannabis’ medical and recreational uses, and its consequent legalization by the different government sectors of developed countries, enhance the development of cannabis worldwide. The plant produces a myriad of structurally and functionally diverse metabolites that play various roles in plant growth and development, and they are effective toxic compounds against herbivores. The production of cannabinoids has been modified for engineering in an in vitro cell-free system [94]. However, cannabis and cannabinoid products have evolved in response to the knowledge of profits from the phytocannabinoid system. They are highly complex owing to decades of use and abuse.

Phytocannabinoids are being investigated for their effect based on therapeutics in brain pathology. Several studies reported in previous databases on human tissues and animal models have highlighted the promising therapeutic potential of cannabinoids in different neurological disorders, including Parkinson’s and Alzheimer’s disease, and the mechanisms of action behind the reported improvement in the clinical outcome and disease progression are associated with their anti-inflammatory, immunosuppressive, antioxidant, and neuroprotective properties, due to the modulation of the endocannabinoid system [94]. Another important molecular feature found in neurodegenerative diseases is the failure of protein homeostasis mechanisms, resulting in undesirable aggregation of misfolded proteins [33]. In this context, CBD has exerted its protective effect over several signaling cascades involved with proteostasis, consequently reducing oxidative stress in cells [94]. The treatments are based on the pharmacophore of existing cannabinoids and treating the spasticity caused by multiple sclerosis in the USA, Canada, and Australia. Its contents, which include CBD and THC, have received regulatory approval in several European countries; however, CBD is a single therapeutic agent. Therefore, clinical studies are ongoing to confirm the efficacy of CBD [95]. Epidiolex (CBD) is in clinical trials for the treatment of resistance seizure disorders, such as Lennox-Gastaut and Dravet syndrome [96]. Based on clinical efficacy and regulation, it also appears to involve the receptor [96]. Phytocannabinoids and cannabinoids, which are effective and safe, are classed as drugs in clinical trials with drugs that fail to demonstrate their efficiency in vivo models. The anticipated legalization at the US federal level and the rapid development of legal cannabis production have led to a strong demand to incorporate cannabis and phytocannabinoids into medicine because of their benefits and low-level side effects on organic cognitive function [85]. With industry support and alternative medicinal research, cannabis is capable of producing an impressive number of cannabinoids exceeding 30% of the flower, bud, and dry weight [97]. Rivaling plant efficacy with microbial fermentation for producing phytocannabinoids such as THC and CBD is challenging, particularly as most significant resources have been put into cannabis agronomy research; in addition, the product sale prices for phytocannabinoids dropped steadily from 2019 to 2021 [5,98]. Because of the numerous potential benefits in the treatment of neurodegenerative disease and other known major diseases [99], synthetic cannabinoid analogs with the potential to exploit cannabinoid treatment without evoking undesirable psychotropic properties are highly desired and may advance research in this area [95]. 

## 10. Conclusions and Future Perspectives

Phytocannabinoids are bioactive natural products found in some flowering plants, liverworts, and fungi that can be beneficial for the treatment of humans and animals and present potent antibiotic effects; most uses are based on anti-inflammatory, neuroprotective, and anti-nociceptive activities. Numerous cannabinoids exhibit promising non-hallucinogenic bioactivities that are variable in nature owing to the side chain and prenyl group and are involved in the biosynthesis pathways. The most important phytocannabinoids possess therapeutic, antibacterial, and antimicrobial properties; thus, they are used in treating several human diseases, and these compounds can contribute to cold, heat, and UV radiation tolerance [100]. Phytocannabinoid biosynthesis can have a constructed nature, and it is used as the foundation of ornamental cannabis research by overcoming certain legal barriers. It has been introduced to the ornamental sector to further evaluate horticultural and cannabis-related research. The bioactive molecules show potential and are well suited to the novel treatment of various diseases in animals [101]. In the meantime, further studies are required for the acceptance of clinical uses for phytocannbinoids and other cannabis-related entities in the context of infectious diseases and antimicrobial activity. Various types of cannabinoids can be produced once additional biosynthetic engineering techniques have been elucidated and a large collection of enzyme molecules is established. However, mere determination of the influence of the abiotic factors on phytocannabinoids production is insufficient [98]. Hemp products, CBD, and THC, are the greatest wholesale potential markets and are highly attractive for their recreational and medicinal properties. However, the ideal extraction technology is expected to be cannabinoid-specific and selective toward the extraction of the CBD compounds [101].

Cannabis may improve plant performance in disease-resistant plants, which can increase the overall content of phytocannabinoids [102,103]. As individual plants are likely to be metabolically active in their cannabinoid formation, phytocannabinoids have versatile uses and beneficial effects. However, any type of cannabinoid can be produced once additional biosynthetic pathways have been elucidated [104,105]. The fate and durability of non-cannabinoids and cannabinoids will be determined by the therapeutic development of numerous rare and novel cannabinoids [106,107]. Synthetic and semi-synthetic cannabinoids are very important for the economy owing to demands from the wholesale market and various cannabis industries to enhance agri-business and prevent plant pathogens [108]. The hypothesis is that phytocannabinoids have versatile use and are beneficial for humans and plants if appropriately used [109,110]. Further investigations are needed on the genes and enzymes involved in the biosynthetic pathways in different plant species [111,112]. The main aim of the review focused on understanding phytocannabinoids through biosynthesis, structural elucidation, and the role of bioactivity in animals as well as whether they possess antimicrobial properties that are important in therapeutics [113,114]. In addition, further investigation is needed on genes and the enzymes involved in the biosynthetic pathways in various sources of plant species [115,116]. The activities of phytocannabinoids play a crucial role in their beneficial effects on animals and humans and provide knowledge that acts as a barrier to the acceptance and utility of cannabis-based antimicrobial therapeutics [117,118]. The physiological activity of cannabis has been largely restricted to THC and CBD compounds; however, some studies have reported that some of the effects arise from the other cannabinoids. Future research should focus on investigating these phytocannabinoids as a key to understanding their limitations. Moreover, information regarding their structural prediction is currently insufficient. The future use of these phytocannabinoids, including their safety in therapeutics, adverse effects, and economic demands, must be addressed. The production of this plant, which is beneficial to animal health, has no negative impact on the environment.

## Figures and Tables

**Table 1:** The Enzymatic Reaction in the biosynthesis of phytocannabinoids.

Biosynthesis	Enzyme	Resulting Product	References
Precursor supply	Olivetol synthase	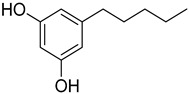	[41]
Type III PKS	Olivetolic acid cyclase	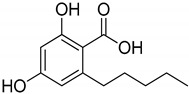	[42]
Tetraketidase cyclase	Cannabigerolic acid synthase	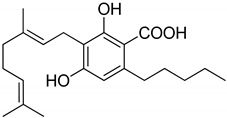	[43]
C-Prenyltransferase	Cannabichromenic acid synthase	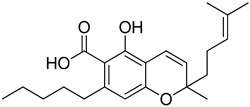	[44]
Oxidocyclase	Cannabidiolic acid synthase	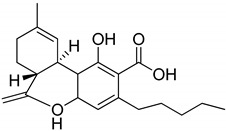	[45]
	Tetrahydrocannabinolic acid synthase	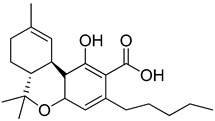	[46]

**Table 2:** Bioactivity and effect of different phytocannabinoids in animals.

Phytocannabinoids	Bioactivity in Animals	References
∆^9^-THC	Pleiotropic effects such as analgesia, muscle relaxation, increased weight and appetite; spasticity, dysphoria	[58]
∆^9^-THCA	Neuroprotective and antitumor activity	[59]
∆^9^-THCV	Non-psychotropic effect on obesity and metabolic disorder	[60]
CBC	Non-psychotropic and anti-inflammatory activity	[61]
CBD	Cures memory loss, obesity, rheumatoid arthritis, epilepsy	[62]
CBG	Non-psychotropic effect	[60]
CBL and DCA	HIV and cancer and boost the immune activity	[63]
CBL	Anti-inflammatory, antimicrobial activity	[64]
-cis-perrottetinene (*cis*-PET)	Increased analgesia, catalepsy, hyperlocomotion, and hypothermia	[65]

## Supplementary Materials

The following supporting information can be downloaded at: https://www.mdpi.com/article/10.3390/metabo13030442/s1, Table S1: Predicted nucleotide composition among tetrahydrocanna-binolic acid (THCA) and Cannabidiolic acid (CBDA) synthase using BioEdit software.
