# Supplementary material for: Biosynthesis of Phytocannabinoids and Structural Insights: A Review
Source: Metabolites. 2023 Mar 17;13(3):442. doi: 10.3390/metabo13030442 (PMC10051821; doi:10.3390/metabo13030442)
Supplement: Supplementary file 1 [file metabolites-13-00442-s001.zip › metabolites-2159432-supplementary.pdf]

Table S1. Predicted nucleotide composition among tetrahydrocannabinolic acid (THCA) and Cannabidiolic acid (CBDA) synthase using BioEdit software.

**Accession**		**MW382908.1**	**MW429550.1**
Gene		Tetrahydrocannabinolic acid synthase	Cannabidiolic acid synthase
Length (bp)		1638	1635
SSMW		495687	495101
DSMW		992139	990612
Nucleotide	A	534	522
	C	282	296
	G	323	325
	T	499	492
G+C content		36.94	37.98
A+T content		63.06	62.02

Note: SSMW: Single strand molecular weight; DSMW: Double strand molecular weight; bp: Base pair.
